# Gastric Cancer Stem Cells Effect on Th17/Treg Balance; A Bench to Beside Perspective

**DOI:** 10.3389/fonc.2019.00226

**Published:** 2019-04-05

**Authors:** Alaleh Rezalotfi, Elmira Ahmadian, Hossein Aazami, Ghasem Solgi, Marzieh Ebrahimi

**Affiliations:** ^1^Department of Immunology, School of Medicine, Hamadan University of Medical Sciences, Hamadan, Iran; ^2^Department of Stem Cells and Developmental Biology, Cell Science Research Center, Royan Institute for Stem Cell Biology and Technology, ACECR, Tehran, Iran; ^3^Faculty of Biological Sciences and Technology, Department of Animal Sciences, Shahid Beheshti University, Tehran, Iran; ^4^Metabolic Disorders Research Center, Endocrinology and Metabolism Molecular-Cellular Sciences Institute, Tehran University of Medical Sciences, Tehran, Iran; ^5^Students Scientific Research Center, Tehran University of Medical Sciences, Tehran, Iran

**Keywords:** gastric cancer, gastric cancer stem cells, Treg, Th17 plasticity, cancer immunotherapy

## Abstract

Gastric cancer stem cells (GCSCs), a small population among tumor cells, are responsible for tumor initiation, development, metastasis, and recurrence. They play a crucial role in immune evasion, immunomodulation, and impairment of effector immunity and believed to be emerged to change the balance of the immune system, importantly CD4^+^ T cells in the chronic inflamed tumor site. However, different subtypes of innate and adaptive immune cells are involved in the formation of the immune system in the tumor microenvironment, we would look at T cells in this study. Tumor microenvironment induces differentiation of CD4^+^ T cells into different subsets of T cells, mainly suppressive regulatory T cells (Treg), and T helper 17 (Th17) cells, although their exact role in tumor immunity is still under debate depending on tumor types and stages. Counterbalance between Th17 and Treg cells in the gastrointestinal system result in the homeostasis and normal function of the immune system, particularly mucosal immunity. Recent data demonstrated a high infiltration of Th17 and Treg cells into the gastric tumor site and proved that tumor microenvironment might disturb the balance between Th17 and Treg. It is possible to assume an association between activation of CSCs which contribute to metastasis in late stages, and the imbalanced Th17/Treg cells observed in advanced gastric cancer patients. This review intends to clarify the importance of gastric tumor microenvironment specifically CSCs in relation to Th17/Tregs balance firstly and to highlight the relevance of imbalanced Th17/Treg subsets in determining the stages and behavior of the tumor secondly. Finally, the present study suggests a clinical approach looking at the plasticity of T cells with a focus on Th17 as a promising dedicated arm in cancer immunotherapy.

## Introduction

Gastric cancer with a high prevalence is the fourth common cancer and second death leading cancer worldwide. About 90% of stomach tumors are adenocarcinomas, which are subdivided into two main histological types: undifferentiated or diffuse type, and well-differentiated or intestinal type, that respectively accounts for 28 and 23% of following lung and liver cancers. In addition to hereditary cases which account for 1–3% of all gastric cancers, environmental factors consist of low consumption of fruits and vegetables and high intake of salts, nitrates, and pickled foods, as well as smoking, gastro-esophageal reflux disease, and obesity have been clearly related to increased risk of gastric cancer.

Epstein-Barr virus and Helicobacter pylori (*H. pylori*) infection cause to 80% of gastric stromal carcinoma and 70% of all gastric cancer cases worldwide (1), respectively. These indicated the important role of the immune system in gastric cancer initiation and development. Indeed, oxidative and nitrosative stress and consequent cellular and DNA damage followed by cycles of repair have been considered as important chain events in *H. pylori*-induced gastric carcinogenesis. Many of these events occur in chronically inflamed gastric mucosa. It is reported that the number of macrophages and dendritic cells (DCs) are increased in the infected gastric mucosa and caused to produce IL-6, IL-1β, IL-12, tumor necrosis factor-α (TNF-α), and stromal cell-derived factor (SDF1α) that induce inflammation and initiation of Th1 responses. Th1 secrets IFN-γ and promotes chronic gastric inflammation. These consecutive events result in an epithelial to mesenchymal transition (EMT) and neoplastic transformation (1). Despite induction of immune response in infected individuals, *H. pylori* evades from adaptive immune response using virulent factors and subverts gastric epithelial cells which in turn mediates inhibition of T cell proliferation and induces Treg cells from naïve T cells. To this gastric epithelial cells express a high level of B7.H1 (PD-L1) (a T cell co-inhibitory molecule) that its interaction with PD-1 leads to a reduction of T cells activity simultaneously with induction of Treg cells. In addition to Treg cells, other CD4^+^ T cells including Th17 cells contribute to T cell responses in infection induced-immunity. It has been reported that IL-17 secreted by Th17, stimulates gastric epithelial cells to release IL-8, which leads to neutrophils recruitment and enhanced chronic inflammation (2). Chronic inflammation can provide a gradual progression from chronic gastritis to gastric atrophy, intestinal metaplasia, dysplasia that is in favor of gastric cancer promotion (3).In fact, *H. pylori* infection induces Th1 and Th17 responses to support chronic inflammation and the unsuccessful clearing of the infection. Moreover, resistance infection stimulates Treg cells to reduce immune response against *H. pylori*. All of these changes favor cancer progression (4). In addition, a correlation between the increased number of Th17 (5) and Treg (6) cells and course of disease was reported in the previous studies. The main concept of the present review is clarifying the role of tumor microenvironment in Th17 and Tregs induction as well as Th17/Treg balance in gastric cancer. Then we pay more attention to the role of cancer stem cells in changing the balance of Th17/Treg and its clinical perspective.

## The Role of the Microenvironment in Gastric Cancer Development

Tumor microenvironment consists of diverse cell types such as tumor cells, gastric epithelial cells, tumor fibroblasts, cancer stem cells, and components of the innate and adaptive immune system. Each cell not only affects on tumor progression but also modulates immune system locally. Indeed persistent chronic inflammation provokes to damage of gastric epithelial mucosa followed by recruitment of bone marrow-derived cells (BMDCs). BMDCs fusion with local gastric epithelial cells leading to tissue remodeling, transformation, and potentially progression of malignancy (7). Moreover, fibroblasts gradually recruit from bone marrow to stomach in response to produced TGF-β and SDF-1α following the inflammation caused by *H. pylori*, to inhibit inflammation and repair the injury, however chronic inflammation derived-dysplasia differentiates them to cancer-associated fibroblasts (CAFs) with the potential to gastric cancer development (8). It has been also observed that CAFs constitute a major stromal compartment actively communicate with cancer cells through growth factors or inflammatory cytokines such as HGF, IL-6, TGF-β, VEGF, FGF, and CXCL12 that can promote tumorigenesis and progression (9). Crosstalk between tumor cells and other stromal cells including MSCs (10), endothelial cells (11), vascular cells, extracellular matrix, tumor-infiltrating lymphocytes (TILs) (12), and tumor-associated macrophages (TAMs) (13) consequently give rise to morphogenesis, angiogenesis, invasion and metastasis of tumor (14), and also modulate the immune system (Table 1). Mechanistically, they act through the cell to cell contact and largely by their secretome including various angiogenic factors, comprising vascular endothelial growth factor (VEGF), interleukin-8, and platelet-derived endothelial cell growth factor (PDGF) in gastric cancer that help tumor progression through escaping the active antitumor immunity (9).

**Table 1:** Cellular components of gastric cancer microenvironment.

	**Cells**	**Function**	**Mediators**	**Reference**
Tumor components	Gastric cancer cells	Induce Treg cells differentiation	TGF-β	(15, 16)
	Gastric epithelial cells	Recruitment of neutrophils and enhanced inflammation	IL-8	(17)
	CD14^+^ gastric cancer mucosa	Mediate FoxP3+ Treg cells infiltration in early stages of gastric cancer	CCL-22 / CCL-17	(6)
	Tumor-derived stromal cells, fibroblasts, and APCs	Recruitment and expansion of Th17 and Treg cells	IL-1β/ IL-6 /IL-23 and TFG-β	(5, 6, 18)
	Gastric epithelial cells	Inhibition of T cell activation	PDL-1	(19)
		Attenuation of MHC-II expression and subsequent antigen presentation	TGF-β	
Host immune system	Neutrophils	Suppress T cells	PD-L1	(20)
		Recruitment of BM-derived cells	IL-6, IL-1β, TNF-α, CXCL-12	(21)
	MDSCs	Deplete arginine and suppress T cells proliferation	Arg-I, iNOS	(22)
	TAMs	Promote the proliferation, invasion, and metastasis of gastric cancer cells	CCL-5	(23)
	Th17 cells	Angiogenesis Tumor progression	IL-17	(24–26)
	Th1 cells	Cause chronic gastric inflammation	IFN-γ	(17)
	FoxP3^+^ Treg cells	Limit Th17 cancer-associated inflammation		(24)

*ALDH, Aldehyde dehydrogenase; CD, Cluster of differentiation; GCSC, Gastric cancer stem cell; CTC, Circulating Tumor Cell; EpCAM, Epithelial cell adhesion molecule; Lgr5m, Leucine Rich Repeat Containing G Protein-Coupled Receptor 5; ABC, ATP-binding cassette*.

Tumor infiltrated immune cells in gastric cancers are included different types of cells such as mast cells, TAMs, and TILs consist of T cells, B cells, and NK cells. The subset of T cells is represented by CD8^+^ cytotoxic T cells, CD4^+^ T helper cells, CD45RO^+^ memory T cells, NK cells, and FOXP3^+^ regulatory T cells. These cells can infiltrate stroma and tumor cells and are considered a manifestation of the host immune response against tumor cells (27).

Clinical studies have indicated that the increased number of Treg cells within TILs may be one of the reasons for insufficient antitumor immunity in cancers (28). The increasing number of Tregs also acts as a tumor promoter in early stages and even in later stages of disease which in turn, can develop progression and metastasis of cancer (29). Moreover, the increased number of Th17 in TILs detected in gastric cancer patients may also involve in gastric cancer development (5).

## Gastric Cancer and Immune Modulation

**Figure 1:**
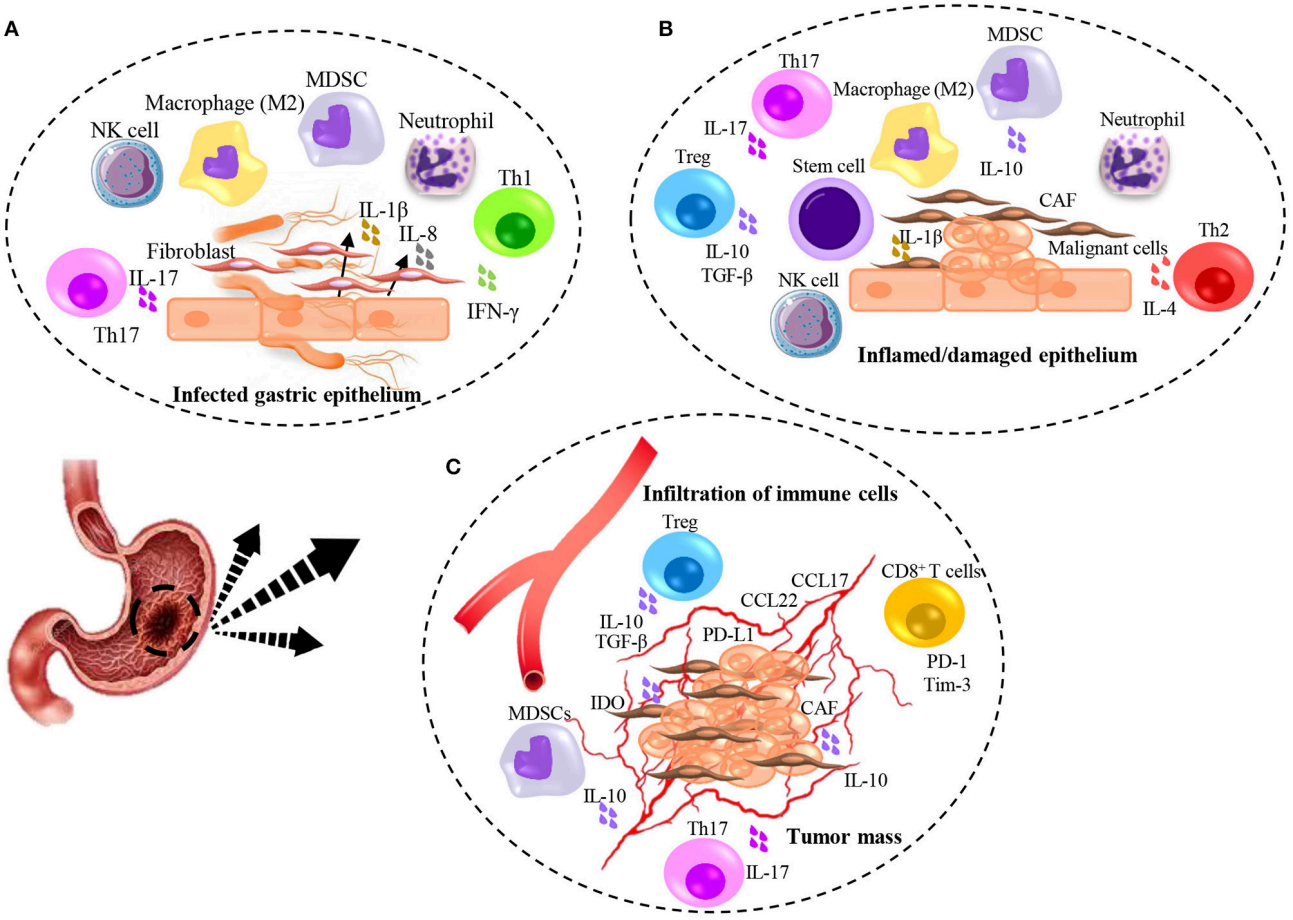
Illumination of immunomodulation induced by H. pylori infection and gastric cancer. **(A)** H. pylori-induced inflammation contributes to initiate damaging of gastric epithelial cells and production of inflammatory cytokines leading to the immediate response of Neutrophils, Macrophages, NK cells, MDSCs, and recruitment of fibroblasts from the bone marrow. Contribution of T cell subsets specifically Th1, result in partial but not complete elimination of bacteria. **(B)** Chronic inflamed/damaged epithelium promotes atrophy. Continued inflammation with the activity of Th1 and Th17 leads to the emergence of Th2 cells that causes tissue remodeling and promotes dysplasia. Production of TGF-β mainly by Treg cells accompanied by IL-6 result in the recruitment of stem cells and differentiation of CAFs which help to initiate the malignancy. **(C)** Tumor angiogenesis contributes to the infiltration of immune cells and promotes tumor growth. The secretion of IL-17 from Th17 cells leads to an increase in the angiogenesis and the further recruitment of suppressor immune cells through chemokines secreted by tumor cells. Further immunosuppressive activity is caused by the expression of co-inhibitory molecules by tumor cells and the secretion of suppressive molecules.

Gastric tumors like other tumors consist of cellular and non-cellular components while their activation promotes initialization, evasion, migration, and progression of cancer. Commonly, tumor cells evade the immune system through downregulation and impairment of the immune responses in malignancies including gastric cancer (Figure 1). One of the critical mechanisms is to interfere and attenuate antigen processing and presentation pathways, leading to the impediment of exposure of neo-antigens (30). Induction of apoptosis through Fas/FasL pathway is the other major mechanism by which tumor cells expressing FasL interact with TILs expressing Fas contributing to Fas-mediated apoptosis (31). Furthermore, immune-suppressor neutrophils expressing PD-L1 activated by tumor-derived granulocyte-macrophage colony-stimulating factor (GM-CSF), have been reported to increase in gastric cancer microenvironment. The activated neutrophils subsequently suppress T cells through engagement of PD-L1: PD-1 inhibitory pathway leads to impairment of antitumor immunity and gastric cancer progression (20). Moreover, TAMs as one of the most frequent infiltrated population in gastric tumor stroma, inhibit antitumor T cell immunity and are related to poor prognosis (31). Macromolecules secreted by fibroblasts (e.g., collagen, fibronectin, and proteoglycan) as extracellular matrix not only shape the tumor and stabilize the physical structure of tumor tissue but also regulate the behavior of infiltrated immune cells inside the tumor microenvironment (32).

Another major component of infiltrated immune cells in the tumor microenvironment are Treg cells. Treg induced anergy contributes to the reversal of recognition and eradication of cancer cells via antitumor T cell immunity. Therefore, Treg cells play a critical role in evading antitumor immunity (30). Interestingly, a unique subpopulation of Treg cells has been identified among infiltrated immune cells in gastric cancer. CD45RA^−^CCR7^−^Treg cells with an effector/memory phenotype, express low level of HLA-DR molecules and accumulated in tumor tissues of patients with gastric cancer. TNF-α produced by tumor cells induces CD45RA^−^CCR7^−^Treg subset and inhibits their HLA-DR expression by phosphorylation and activation of STAT3. This immune suppressive population substantially prevents antitumor CD8^+^ T cells *in vitro*, while supports the tumor growth and progression via IL-10 production as well as cell to cell contact and also is associated with advanced stages of the disease and reduced survival (33).

It has been shown that gastric myofibroblasts (GMFs) that are highly-expressed MHC-II cells can induce Th17 cells differentiation from CD4^+^ T cells under Th17-polarizing condition. The enriched Th17 population in inflammatory milieu might lead to the persistence of inflammation and is associated with carcinogenesis (34).

## Th17 and Treg Recruitment/Expansion by Gastric Cancer Components

CD4^+^CD25^hi+^Foxp3^+^ regulatory T cells are known with their inhibitory activity, largely by production of TGF-β and play a critical role in preventing autoimmunity and tumor progression (35), while IL-17 producing RORγ^+^ Th17 cells have well-described roles in autoimmune disease, although their role in tumor immunity remained unknown (36). As mentioned previously, gastric cancer is a clinopathology state of chronic sterile inflammation that provides an immunosuppressive condition resulting infiltration of high frequencies of both Th17 and Treg infiltration (37). Mechanistically, expression of lymphoid homing receptors including CCR4, CCR6, and also CD62L on Treg cells during cancer development leads to the gradually increased number of tumor-associated Treg cells (38) through secretion of CCL17 and CCL22 (ligands for CCR4) by tumor cells as chemotactic factors in early stages of gastric cancer (39). Indeed, tumor inflammatory milieu intelligently recruits Treg cells to suppress antitumor immunity (18). Most of the stromal cells beside gastric cancer cells have the potential of producing TGF-β followed by activation of hypoxia-inducible factors-1α (HIF-1α) in the tumor microenvironment. This in turn promotes Treg infiltration (15). However, TGF-β alone is not enough to infiltrate Treg (40) and presence of prostaglandin E2 (PGE2) and also H-Ferritin may favor for FOXP3^+^ cells infiltration and differentiation (16).

Although it has been frequently reported that Treg cells are significantly prevalent in gastric cancer, recent evidence has emphasized on the importance of the disturbed balance of Th17 and Treg cells in gastric cancer patients, while the ratio of Th17/Treg is obviously higher in patients with advanced gastric cancer compared to healthy controls. Furthermore, patients with lymph node metastasis have indicated a significantly increased ratio of Th17/Treg cells (24). Tumor-derived fibroblasts produce MCP-1 (CCL2) and RANTES (CCL5) could attract Th17 cells intensively. In the murine immune system, TGF-β, and, IL-6 strongly stimulate Th17 and IL-17^+^CD8^+^ cells synchronously, while exogenous IL-2 significantly reduces both Th17 and IL-17^+^CD8^+^ cells *in vitro* and conversely increases the number of Treg cells. Moreover, the blockade of IL-2 leads to a decrement in number of Tregs, while enhancing IL-17^+^CD4^+^ and IL-17^+^CD8^+^ populations. It can be concluded that IL-2 may have opposite effects on Th17 and Treg differentiation in the murine system. This is indicative of the key role of IL-2 besides TGF-β and IL-6 in the regulation of Th17/Tregs balance (41). Moreover, although Th17 cells differentiation is driven by TGF-β in mice, its role in human remained controversial (42). MDSCs, a population in tumor microenvironment also promote either Treg or Th17 cells expansion by their secretion (43). Most of the cells in tumor microenvironment recruit and expand Treg and Th17 cells through production of cytokines and chemokines (44).

## The Function of Il-17 Producing Cells in Gastric Cancer: A Controversial Story

CD4^+^T cells (Th17) and CD8^+^ IL-17 producing cells T cells (Tc17) have reported in patients with gastric cancer (45). It has been suggested that both IL-17^+^CD4^+^ and IL-17^+^CD8^+^ in tumor microenvironment can take a pathogenic role contributing to tumor progression (41). It has been also depicted that the expression of IL-17 in gastric cancer tissues and an increased number of Th17 might be related to tumor promotion due to IL-17-mediated inflammation (24). Moreover, there is evidence for the positive effect of IL-17 on the production of pro-angiogenic factors including VEGF, prostaglandin E1 (PGE1), PGE2 and macrophage inflammatory protein-2 (MIP-2) by fibroblasts and tumor. In addition, vascular endothelial cell migration and cord formation stimulated by IL-17 leading to increased angiogenesis and promote tumor growth. It has been also dedicated that IL-17 can provoke production of IL-8 in both epithelial cells and macrophages which in turn, may enhance the recruitment of inflammatory cells into the tumor sites. Neutrophils with or without macrophages are activated through IL-8 stimulation, and also have been related to tumor progression [77] by several mechanisms including angiogenesis and invasion (46). These data suggest that IL-17 production by Th17 CD4^+^ cells in tumor microenvironment leads to tumor progression by angiogenesis and neutrophil infiltrating in patients with gastric cancer (25). A novel subpopulation of ex-Th17-FoxP3^+^ cells has been shown to have a substantial role in tumor initiation and progression. This study has reported a dual role for this population. While RORγt expression promotes an inflammatory response, the expression of FoxP3 commits the suppressor actions (47). These data propose a potential role for inflammatory Th17 cells in cancer pathogenesis.

In contrast, some other studies have suggested that increased level of IL-17 in tumor site leads to the improved antitumor immunity of TCD4^+^IL-17^+^ cells through inducing Ag-specific cytotoxic T cells (48), while tumor infiltrated Th17 cells *per se* are not able to kill or inhibit tumor cells proliferation *in vitro* and conversely, promote tumor progression due to the existence of TGF-β and IL-6 in local tumor site (18). In addition, it has been reported that tumor-infiltrating Th17 cells express several effector cytokines in cancer patients, similar to that observed in patients with infectious diseases. This suggests that tumor-associated Th17 cells might also play an antitumor role in the context of the tumor. According to this possibility, Th17 cells are positively associated with effector immune cells, including cytotoxic CD8^+^ T cells, NK cells, and IFNγ-producing Th1 cells in the tumor microenvironment *in vitro* and human. Moreover, it has been documented that tumor growth and lung metastasis enhanced in many IL-17-deficient tumor models, and forced expression of IL-17 in tumor cells was shown to suppress tumor progression (49).

It has been investigated that IFN-γ-producing Th1-like cells, which seemed to be converted from CD8^+^IL-17 producing cells, exhibited strong cytotoxicity for the eradication of tumor cells. This conversion of Tc17 cells into Th1-like cells may be due to epigenetic modifications as seen in Th17 cells, appearing to be critical for the acquisition of the antitumor feature for Tc17 cells in tumor immunity (50). Therefore, depending on the milieu, Th17 cells can accept both pro/antitumor roles in the tumor microenvironment.

## Th17/Treg Plasticity in Gastric Cancer

Different types of T cells including Th1, Th2, Th17, and Treg cells exhibit significant developmental plasticity through epigenetic mechanisms (51) that are required to preserve homeostasis particularly in the gastrointestinal tract (52). The induction of selective gene expression that results in the development of distinct phenotypes, comes from changes in cytokine milieu that can be sensed by the signal transducer of transcription factors which in turn regulate the expression of master regulators of each lineage with the consideration of chromatin accessibility (53).

It has been suggested that FOXP3^+^ Treg cells might become unstable under certain inflammatory conditions and might adopt a phenotype that is more characteristic of effector CD4^+^ T cells (54). In addition, it was argued that loss of FoxP3 expression resulted in the capacity to become IL-17-secreting cells. Furthermore, in response to IL-12 *in vitro*, Treg cells can produce IFN-γ (55).

Th17 cells have also shown the plasticity based on the milieu in which they are located and emerge the transient phenotypes with partially inflammatory and suppressive phenotype. IL-17^+^Foxp3^+^ T cells can be derived from CCR6^+^ but not CCR6^−^ T cells and play role in Th17/Treg differentiation process. They represent partially Th17 (inflammatory) and Treg (inhibitory) cells. Moreover, IL-17^+^Foxp3^+^ T cells, as proinflammatory Treg cells produce IL-17 and moderate levels of IL-2, IFN-γ, and TNF-α resulting in the aggravated inflammatory response (56). Identification of FoxP3^hi^ and FoxP3^lo^-IL-17 producing Tregs is evidence for *de novo* FoxP3 expression in IL-17 producing T cells in human (57). Preclinical studies have implicated phenotypic markers in IL-17^+^Foxp3^+^ T cells overlapping between Treg cells and Th17 cells. This population simultaneously expresses CD25 and CCR4 that identified in Treg cells and CD161 and CD49d as Th17 cell markers (37) as well as Th17/Treg plasticity markers (58). Th17 cells also might be a source of tumor-induced FoxP3^+^ cells besides nTreg and iTreg cells which have developed from naïve CD4^+^ precursors (58). in addition, FoxP3^+^RORγt^+^ IL-17-producing T cells as an unstable lineage have detected in colon cancer (59) proposed that they can be originated from FoxP3^+^Treg cells. This subset has preserved their immune suppressive function, while they have lost their anti-inflammatory function, Therefore regulation of the balance between Th17 and Treg subsets from a common precursor depend on the milieu and it seems that Treg cells enrichment may have a key role in Th17 development (60). Further analysis suggests that impairment of Th17-to-FoxP3^+^ T cells along with induction of FoxP3^+−^to-Th17 (58)-to-Th1-like (61) IFN-γ producing cells transdifferentiation can be a reliable approach for Treg cells depletion within the tumor microenvironment, due to the inability of committed Th1 to convert to Treg in Th1-Th17-Treg axis (55). Studies on autoimmune disorders have shown that ex-FoxP3^+^IL-17^+^ cells are accumulated selectively at the inflammation sites. This is another proof of plasticity feature of FoxP3^+^ T cell subset, whereas the committed Treg cells are stable (62). Metabolic regulator of immune responses such as nutrient, energy, oxygen, and stress level along with transduction signals like mTOR, HIF-1α, and AMP-activated protein kinase (AMPK) regulate the Th17/Treg balance. Therefore, besides inter-conversion developmental factors, additional factors induce the Th17/Treg balance through transcription factors. In this context, the same signal might lead to both Th17 and Treg induction depending on the microenvironment components. For instance, PGE2 increase Th17 through the production of IL-23 and IL-1β by DCs and macrophages, while it can also induce expansion of IL-10 producing Treg type-1 cells (Tr1) as a result of tumor cells secretion as well as expression of COX2/PGE2 by Treg cells (63). Activation of JAK/STAT, TGF-β, STAT3 and mTOR also skew the Th17/Treg balance toward Th17 cells differentiation (64).

Recent studies have demonstrated that tumor-derived Th17 cells produce low levels of TGF-β and IL-10 after stimulation with anti-CD3 *in vitro* and express CTLA4, FoxP3, and CD25 as Treg cell markers, while they do not suppress tumor progression. This in turn, confirms the developmental plasticity of Th17 cells and exhibits a yin and yang performance, meaning that Th17 infiltrating cells have either a regulatory or an antitumor role in gastric cancer microenvironment. Enhanced production of PGE2, IL-1β, IL-6, TGF-β as well as arginase, indoleamine 2,3-dioxygenase (IDO) and IL-10 from MDSCs implicitly mediate reciprocal differentiation of Th17 and Treg cells in a defined circumstance of tumor microenvironment (65). IDO and iNOS produced by MDSCs both are critical molecules for regulation of Th17/Treg balance. Descriptively, it has been investigated that Th17 differentiation induces through IL-6R mediated pathway. In addition, iNOS/NO induces TGF-β mediated FoxP3^+^ Tregs differentiation and downregulates IL-17-mediated Th17 responses (66). Moreover, Helios, a transcription factor involved in FoxP3^+^Treg cells development stability, is also associated with development of highly suppressive Treg cells (67) and is highly expressed in the tumor microenvironment (68) which in turn, might have a role in the regulation of the balance between Treg and Th17.

## Gastric Cancer Stem Cells

Recent findings suggest that CSCs, as immortal tumor-initiating cells with self-renewal property and pluripotent capacity, have been characterized in multiple malignancies including leukemia and different solid tumors. CSCs due to their exceptional features are responsible for tumor initiation, development, metastasis, and recurrence. Based on the CSC model, all other cells within the tumor bulk are derived from primary differentiated CSCs, without considering the existence of mutations and genetic variations during tumor development. This event named “clonal evolution model.” To date, CSCs have been identified in various solid tumors including gastric cancer (69). GCSCs are defined and isolated by cellular markers expression (70) that are listed in Table 2. In addition to cellular markers, a variety of methods are used to identification and isolation of CSCs including side population, sphere formation, *in vivo* tumorigenicity, self-renewal capacity and signaling pathways, although these methods have advantages and disadvantages, and should be used according to the tumor types and tumor location (83). These cells exhibit potential to form tumor spheres under non-adherent cell culture conditions and form gastric tumor xenografts in immune-deficient mice (84), as well as escape from immune-mediated destruction (85). The chronic infection and *H. pylori* contribute to TGF-β1 production that induces gastric cancer stem cells emergence that is in favor of early stages of gastric tumorigenesis and elicits an EMT (86).

**Table 2:** Gastric cancer stem cell markers.

**Markers**	**Sources**			**Main Points**	**Reference**
ALDH ^high/low^,CD133, CD44	Sphere	Mouse Forestomach Carcinoma (MFC)		-ALDH^high^ GCSCs possess a high level of self-renewal ability but resting stage. -The ALDH^low^ GCSCs with limited self-renewal ability, but a rapid proliferation stage.	(71)
CD24, CD44, Vimentin, ALDH, Cytokeratin 18^low^	Sphere	MKN-45, SGC7901		MKN45 cells exhibited a higher sphere-forming efficiency than SGC7901 cells with higher expression of CD44 and CD24.	(72)
CD44, CD133, CD24	Sphere	Tumor tissue		-Primary cancer tissues express less CD44 and CD133 compared with metastatic cancer tissues. -CD44 increases Oct4 expression through ERK pathway in a positive feedback loop and maintains the stemness of gastric cancers. -CD44 might be a driving factor in the development of CSCs in addition to being a surface marker.	(73, 74)
CD44, CD54	Sphere	Tumor tissue Circulating GCSC		-GCSCs are indeed in the circulation and support the hypothesis that the CTC population contains CSCs.	(75)
EpCAM, CD44	Sphere	Tumor tissue		-Both CD44 and EpCAM markers are needed for isolation of cancer stem cells directly from patients. -In addition to *in vivo* experiments, gastric cancer stem cells generate various differentiated cells in cancer sphere culture.	(76)
CD90	Primary tumor cells	Tumor tissue Xenotransplantation		A higher proportion of CD90^+^ cells correlates with higher *in vivo* tumorigenicity of gastric primary tumor models.	(77)
EPCAM, CD133, CD166, CD44, ALDH^high^	Primary tumor cells Cell line Cell line	Xenograft MKN-45, MKN-74	Sphere Sphere	-CD44 and ALDH are the most specific biomarkers to detect and isolate tumorigenic and chemoresistant gastric CSCs in noncardia gastric carcinomas. -Tumorigenic and chemoresistant gastric CSCs co-express EPCAM, CD133, CD166, CD44, and ALDH.-ALDH is the most specific biomarker for CSC enrichment before CD44 in both diffuse- and intestinal-type noncardia gastric carcinomas.	(78)
EpCAM, CD44, CD44v8-10, CD133	Primary tumor Primary tumor	Xenograft Tumor tissue		Unlike CD44s that is expressed in many normal tissues, CD44v8-10 marks human gastric CSCs and contributes to tumor initiation, possibly through enhancing oxidative stress defense.	(79)
CD71	Cell line	MKN-1		CD71^−^ cells have important roles in cancer development. This subtype also exhibits high drug resistance to conventional chemotherapy.	(80)
Lgr5	Human gastric cancer and animal model	Tumor tissue		Gastric cancer develops when cancer-associated genes are activated in Lgr5-positive stem cells and change them to CSCs.	(81)
ABCB1, ABCG2, CD133	Human gastric cancer Different differentiation status cell lines Xenograft transplantation of 3 cell lines	Tumor tissue HGC-27, BGC-823, SGC-7901 Injected HGC-27, BGC-823, SGC-7901 cell lines		-The expression of the CSC markers ABCB1, ABCG2, and CD133 differ in gastric cancers with various degrees of differentiation. -Poorly differentiated gastric cancers expressing relatively more CSC markers.	(82)

CSCs similar to other tumor cells evade the immune system by changing their immunogenicity and also are capable to impair the immune response through the expression or secretion of factors impeding antitumor immune responses. Interestingly, CSCs are also able to partially mimic antigen presenting cells (APCs) with regard to MHC I and PD-L1 expressions. Elevated expression of PDL-1 on CSCs surface, inhibit T cell activation and induce anergy. CSCs secret TGF-β in more concentration than their non-CSCs. TGF-β secretion is associated with decreased expression of MHC II and subsequently attenuate antigen presentation, whereas it stimulates regulatory T cells expansion (87).

Several studies have identified a connection between innate immune cells (i.e., macrophages and MDSCs and DCs) and adaptive immune cells such as regulatory T cells with CSC. The immune cells could accelerate CSC-specific expansion and maintenance both directly and indirectly via their secretions. The interaction between Treg cells and CSCs largely remained obscure, but a recent study concerning the role of Treg cells in colorectal cancer has been proved that FoxP3^+^IL-17^+^ cells promote the expansion of CSCs by secreting of hypoxia-induced IL-17 (30).

## Th17/Treg Plasticity Can be Possibly More Affected by Gastric Cancer Stem Cells

Although the polarization of Treg vs. Th17 cells via interaction with MSCs is indicated (88), the exact role of CSCs particularly GCSCs on Th17/Treg plasticity remained to be determined.

Soluble factors and cell to cell contacts are two main factors from GCSCs can affect Th17 differentiation.

### Soluble Mediators in Th17/Treg Polarization by GCSCs

**Figure 2:**
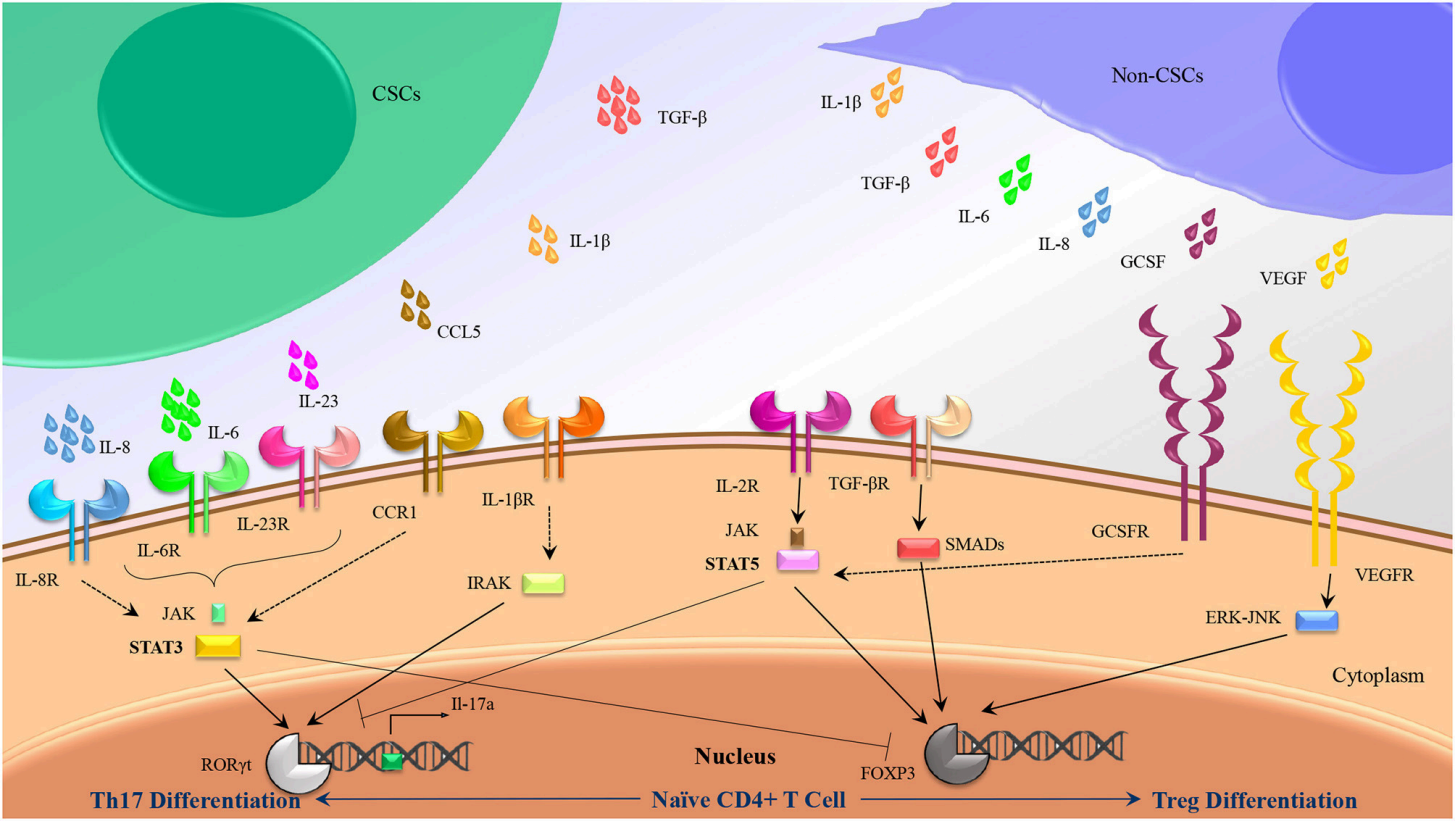
Th17/Treg polarization might be influenced by CSC secretions rather than non-CSC secretions. Besides intrinsic mediators associated to Th17 (IL-1β, IL-6, and Il-23) and Treg (TGF-β, IL-2) differentiation, some other cytokines produced by CSCs and non-CSCs might induce Th17 and Treg differentiation by crosstalk with the similar internal adaptors.

As we stated previously, accumulating data have indicated that a cocktail of IL-6, IL-1β, and IL-23 could be used as dedicated cytokines for induction of Th17, whereas IL-2 and TGF-β have been frequently considered as stimulating factors in human Treg cells differentiation. Further analysis has suggested a dual role for CSCs to induce Treg and Th17 subsets in several cancers through alteration in cytokines balance in tumor milieu (Figure 2).

Despite intensive researches on CSCs in various cancers, there is no evidence regarding the GCSCs effect on Th17/Treg balance. Most of the evidence came from malignant tumors with a higher incidence of GCSCs. High plasma levels of platelet microparticles (PMP), VEGF, IL-6, and CCL5 in patients with stage IV vs. those in patients with stage I or stage II/III that can be related to metastatic gastric cancer (89) and gastric cancer stem cells activation.

IL-6 that is important in Th17 differentiation, as well as cancer-associated inflammation, was detected in oral squamous cell carcinoma (90) and *in vitro* culture of KM22, a breast cancer cell line (91). Alternatively, it was determined that IL-6 is produced by CSCs in multiple myeloma, breast cancer (22), and Squamous cell carcinoma with a relatively significant concentration (92) rather than other cancer cells. Similarly, IL-23 produced by ovarian CSCs (93) has been frequently reported that plays an important role in maturation and maintenance of Th17 cells phenotype. Of note, TGF-β a critical cytokine for induction of Treg cells and inhibition of Th17 cells differentiation has been detected in adenocarcinoma, squamous cell carcinoma and lung cancer cell lines (94), although it was produced in more concentration by CSCs in squamous cell carcinoma (92). In addition, CCL5 a chemokine that strongly attracts Th17 and Treg cells has been detected in breast cancer at primary and metastatic tumor sites, ovarian, gastric, and prostate cancers (95). Accordingly, some other cytokines produced by tumor mass might induce Th17 and Treg differentiation by crosstalk with the similar internal adaptors. IL-8 produced by many types of cancers (94) has been demonstrated that significantly secreted by squamous cell carcinoma CSCs (92), and it can trigger differentiation of Th17 cells through simultaneous activation of STAT3 with IL-6. Similarly, VEGF produced by MKN-45 as a gastric cancer cell line (96), might induce Treg differentiation through ERK-JNK signaling pathway activation (97, 98).

### Direct Communication Between GCSCs and T Cells

In addition to soluble factors secreted by CSCs that have effect on Th17/Treg balance, existence of stemness genes in Th17 cells (it will be discussed in part 5) can potentiate this hypothesis that they can transfer between CSCs and CD4^+^ T cells through cell to cell communication. This in turn, can induce Th17 cells differentiation. Diverse mechanisms of intercellular communication have been already well documented including chemical synapses, gap junctions, and plasmodesmata. High sensitive nanotubular structures can be established probably between immune cells, tumor cells, and also between infiltrating T cells and distant tumor cells, facilitate simplify selective transfer and communication (99). Thereupon, stemness genes might transfer from CSCs to T cells through tunneling nanotubes and differentiate CD4+ T cells to long-lived Th17 stem-like cells. STAT3, a pleiotropic transcription factor activated downstream of cytokines, is overexpressed in gastric cancer stem cells and metastatic tumor samples (100). Activated STAT3 can be either pro-oncogenic or tumor-suppressive according to the tumor etiology and mutational landscape (101), and it considers as a critical transcription factor for Th17 differentiation, while represses the development of Tregs (102). Therefore, we can conclude that STAT3 might be able to pass through membrane structures between gastric cancer stem cells and Treg/CD4^+^ uncommitted T cells to change the shift them to Th17 cells.

The tumor's immune cell polarization changes are basically beneficial to the tumor, leading to escape from the immune system and tumor progression. However, the antitumor or protumor activity of Th17 cells induced by GCSCs should be investigated.

## Future Prospective

While CSCs are a small population among tumor cells, their importance in immune modulation should not be neglected. These cells produced a high concentration of soluble factors that differ from other tumor cells, therefore determine the fate of Th17 and Treg cells by changing in differentiation, recruitment. Thus, the imbalanced Th17/Treg in peripheral blood and tumor tissues could be considered as new hallmarks of CSCs activity and metastasis.

Recently, cancer immunotherapy aims to elicit the activity of CTLs within a tumor, strengthening the helper CD4^+^ T cells function can improve the efficiency of antitumor activity of CTLs, clonal expansion, and providing effector and memory CTL (103). Although most researches have focused on immune activation using T CD8+ and even CD4+, the exact role of Th17 and its potential in immune cell therapy remained unknown. In the present study, we reviewed the characteristic of Th17 cells, as they are not fully differentiated subset with self-renewal ability, sustained survival, capable of plasticity, and representing stem cell-like memory cells features. They have been reported as a precursor of Th1 cell-like cells producing IFN-γ and CD8^+^ cytotoxic cells which play an important role in antitumor immunity (104). Hence, they could be considered to be promising for using in cancer immunotherapy strategies. Two strategies including:

### Conversion of FoxP3^+^CD4^+^Treg Cells to the Hybrid Th17/Th1 to Elicit a Potent and Prolonged Antitumor Immune Response

In contrast to Th17, Th1 as a key player in antitumor immunity possessing functional mediators including IFN-γ^hi^, CD107a^hi^, T-bet^hi^, and Granzyme-B^hi^ as markers of activities are not able to persist in tumor microenvironment to show a long-term effect probably due to the lack of stem cell features. Recent data has been shown that KLF4, a key transcription factor in pluripotency of stem cells, binding to the promoter of *Il17a* plays a critical role in Th17 differentiation but not in other subsets of helper T cells (105). Moreover, the long-lived Th17 cells with the capacity of plasticity, exhibit an increase in expression of genes associated with self-renewal including HIF-1a, Notch, Bcl2, OCT4, and Nanog (106). In addition, Th17 cells do not express PD-1, FoxP3, KLRG-1, CD57, and IL-10, therefore they are not a candidate for being functionally exhausted PD-1^+^ T cells, suppressive Foxp3^+^, IL-10^+^ T cells or senescent CD28^−^CD57^+^KLRG-1^+^ T cells (106).

We propose an approach to convert CD4^+^FoxP3^+^ cells isolated from patients to transient ex-FoxP3^+^Th17 cells. Th17 cells have been observed to be able to switch to ex-Th17IFN-γ^+^ from IFN-γ^+^IL-17^+^ cells in autoimmune diseases and inflammatory infections (50). Therefore, the next step would be the differentiation of Th17/Th1 as desired cells with an antitumor activity by providing proper cytokine cocktail. We suggest that the combination of the potent Th1 effectiveness with the stemness features of Th17 to produce hybrid Th17/Th1 in human and mouse can be more efficient to control tumor progression compared to Th1 or Th17 alone as previously reported in melanoma. The hybrid Th17/Th1 has exhibited a potent effector function and an increased persistence with less susceptibility to induced cell death through activity (107). Although it might be more challenging to identify and keep the hybrid phenotype.

### Production of Tc17/CTL Population From FoxP3^+^CD8^+^Treg Cells With Higher Potential of Antitumor Immunity

It has been documented that Tc17 cells show no strong cytotoxicity, whereas plastically changed IL-17/IFN-γ cells through epigenetic modification (108) have a strong antitumor effect. Cultivation of Tc17 cells with further IL-12 convert them to IL-17/IFN-γ double producing cells with acquired cytotoxic function *in vitro* and *in vivo* (50). Therefore, we propose a strategy in order to convert CD8^+^FoxP3^+^ cells into ex-Foxp3^+^Tc17 cells and then into the Tc17/CTL population which plays a crucial role as final effector cells with cytotoxicity in the tumor microenvironment.

**Figure 3:**
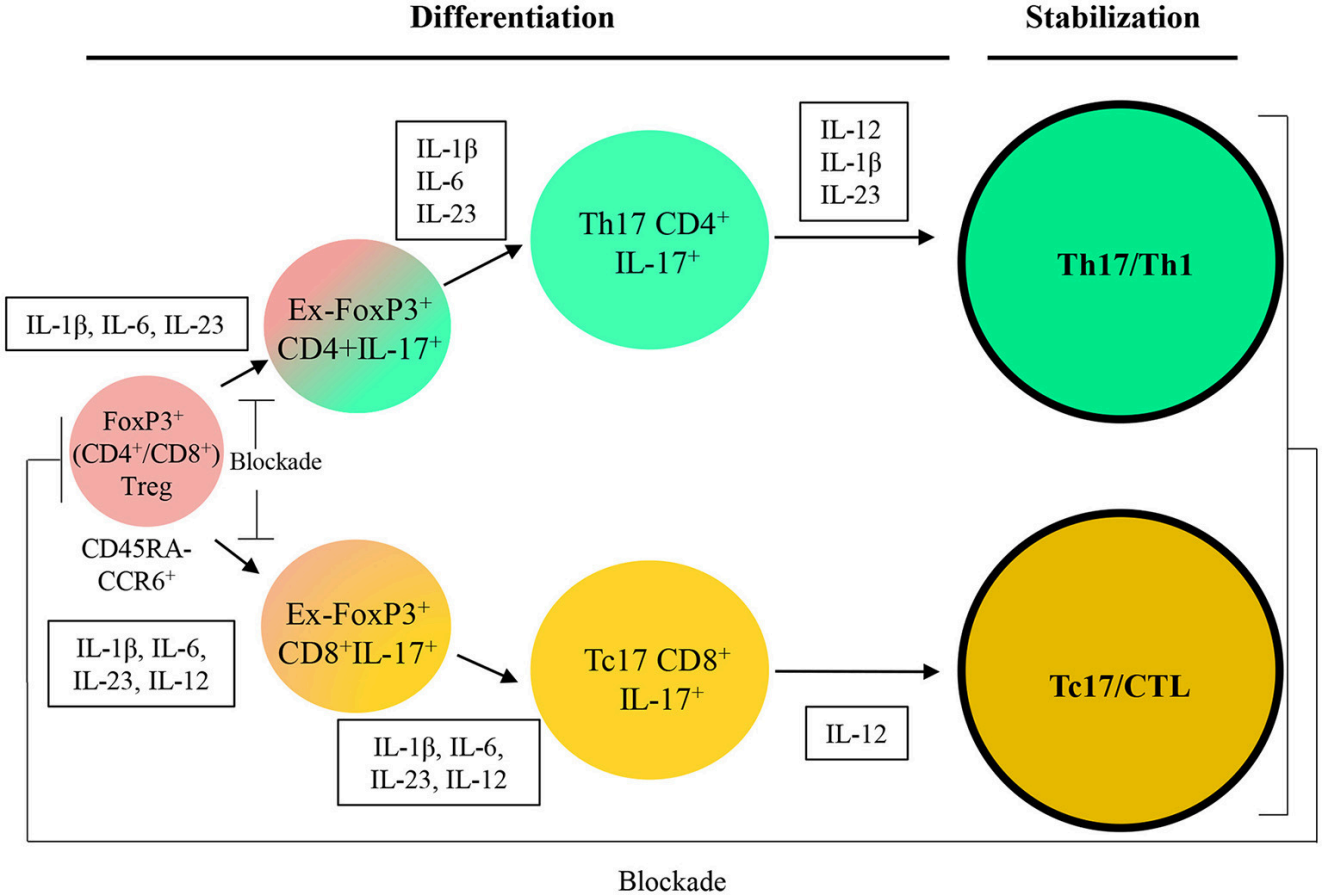
A therapeutic approach based on Treg/Th17/Th1 axis. IL, Interleukin*; CCL*, C-C motif chemokine; CXCL, Chemokine (C-X-C motif) ligand; APC, Antigen-presenting cells; TGF-β, *Transforming growth factor beta;* PDL-1, Programmed death-ligand 1; MHC, *Major histocompatibility complex*; MDSC, Myeloid-derived suppressor cell; Arg-I, Arginase I; iNOS, Inducible nitric oxide synthase; TAM, *Tumor*-associated macrophage; IFN-γ, Interferon-gamma.

Further attempt for the two strategies is would be focusing on stabilization of induced (reactivated) antitumor immune cells by keeping the one-way conversion of inefficient to efficient cells as well as blocking internal pathways to unfavored cell fates (Figure 3). We believe that recent strategies are able to bring new insight to apply T cells plasticity in cancer immunotherapy and suggest that this feature can be used as a promising approach in the treatment of cancers and also autoimmune diseases.

## Conclusion

Accumulating evidence suggests that CSCs including GCSCs can have a greater effect on Th17/Treg balance than other tumor cells. Therefore, the observation of imbalanced Th17/Treg in liquid biopsy of cancer patient's blood could be considered as a diagnostic marker for activation of CSCs. In addition, a change in the axis of Th17/Treg indicates that CSCs alter the balance between them through the plasticity of T cells with the focus on Th17 plasticity. Therefore, this feature can be an opportunity and uses in immunotherapy of cancers to convert the patient's inefficient cells into active and antitumor cells to remove the tumor.

## Author Contributions

AR contributed to develop the theory, data collection, and was the major contributor in writing the manuscript. HA contributed to data collection and data analysis. EA was involved in data collection, drafting and writing the manuscript. GS revised the manuscript and supervised this work. ME developed the theory, revised the manuscript, and supervised this work. All authors read and approved the final manuscript.

### Conflict of Interest Statement

The authors declare that the research was conducted in the absence of any commercial or financial relationships that could be construed as a potential conflict of interest.

## Abbreviations

Th: T helper
Treg: Regulatory T cell
MHC: Major histocompatibility complex
TGF-β: Transforming growth factor beta
IL: Interleukin
IFN-γ: Interferon gamma
CD: Cluster of differentiation
H. pylori: Helicobacter pylori
EMT: Epithelial to mesenchymal transition
CCL: Motif chemokine
CXCL: Chemokine (C-X-C motif) ligand
DCs: Dendritic cells
TNF-α: Tumor necrosis factor-α
SDF-1α: Stromal cell-derived factor-1α
MDSCs: Myeloid-derived suppressor cells
Arg-I: Arginase I
iNOS: Inducible nitric oxide synthase
PDL-1: Programmed death-ligand 1
BMDCs: Marrow-derived cells
TAMs: Tumor associated macrophages
CAFs: Cancer-associated fibroblasts
APCs: Antigen presenting cells
TILs: Tumor infiltrating lymphocytes
GMFs: Gastric myofibroblasts
MCP-1: Monocyte chemoattractant protein-1
RANTES, Regulated on activation: normal T cell expressed and secreted
PGE2: Prostaglandin E2
PGE1: Prostaglandin E1
HIF-1α: Hypoxia-inducible factors-1α
VEGF: Vascular endothelial growth factor
MIP-2: Macrophage inflammatory protein-2
IDO, Indoleamine 2: 3-dioxygenase
PMP: Platelet microparticles
CpG-ODNs: CpG oligodeoxynucleotides
pDCs: Plasmacytoid dendritic cells
TLR-9: Toll-like receptor 9.
